# Urine Complement Factor Ba Is Associated with AKI in Critically Ill Children

**DOI:** 10.34067/KID.0000000000000077

**Published:** 2023-02-09

**Authors:** Erin K. Stenson, Charles L. Edelstein, Zhiying You, Shinobu Miyazaki-Anzai, Joshua M. Thurman, Bradley P. Dixon, Michael Zappitelli, Stuart L. Goldstein, Ayse Akcan Arikan, Jessica Kendrick

**Affiliations:** 1 Section of Pediatric Critical Care Medicine, Department of Pediatrics, University of Colorado School of Medicine, Aurora, Colorado; 2 Division of Renal Disease and Hypertension, Department of Medicine, University of Colorado School of Medicine, Aurora, Colorado; 3 Renal Section, Department of Pediatrics, University of Colorado School of Medicine, Aurora, Colorado; 4 Division of Paediatric Nephrology, Department of Pediatrics, The Hospital for Sick Children, Toronto, Ontario, Canada; 5 Center for Acute Care Nephrology, Department of Pediatrics, Cincinnati Children's Hospital Medical Center, Cincinnati, Ohio; 6 Divisions of Pediatric Critical Care and Renal, Department of Pediatrics, Baylor College of Medicine, Texas Children's Hospital, Houston, Texas

**Keywords:** Acute Kidney Injury and ICU Nephrology, acute kidney injury, complement, factor B, pediatric intensive care medicine, pediatric nephrology

## Abstract

**Key Points:**

- Complement activation, specifically factor B, is implicated in AKI pathogenesis in animal models.
- Urine Ba (an activation fragment of factor B) was significantly higher in critically ill children with stage 3 AKI and sepsis-AKI.
- If larger studies show similar association between urine Ba and AKI severity, clinical trials of factor B inhibition are warranted.

**Background:**

Critically ill children with AKI have high morbidity and mortality rates and lack treatment options. Complement activation is implicated in AKI pathogenesis, which could be treated with complement-targeted therapeutics. We assessed for an association between urine Ba, an activation fragment of the alternative complement pathway, and AKI in a large cohort of critically ill children.

**Methods:**

A biorepository of children requiring mechanical ventilation was leveraged. AKI was based on pediatric version of the RIFLE criteria—stage 1: 25% decreased eGFR or urine output (UOP) <0.5ml/kg per hour for 8 hours; stage 2: 50% decreased eGFR or UOP <0.5 ml/kg per hour for 16 hours; stage 3: 75% decreased eGFR or UOP <0.3ml/kg per hour for 24 hours or anuric for 12 hours. ELISAs were performed to quantitate urine Ba values. Log Ba was used in ANOVA with pairwise comparison by the Tukey method. Logistic regression was performed to test the association between urine Ba and AKI diagnosis.

**Results:**

Seventy-three patients were included, of which 56 had AKI: 26 (46%) stage 1, 16 (29%) stage 2, and 14 (25%) stage 3. Ba was significantly higher in patients with stage 3 AKI compared with all other stages. Ba was higher in sepsis-associated AKI compared with non–sepsis-associated AKI. Multivariate analysis included urine Ba, urine IL-18, urine NGAL, sepsis, and Pediatric Risk of Mortality Scores-II (an estimate of illness severity) and showed a significant association between urine Ba and AKI (odds ratio 1.57, 95% confidence interval, 1.13 to 2.20; *P* 0.007).

**Conclusion:**

Urine Ba is significantly increased in patients with AKI compared with patients without AKI. In patients with similar illness severity, a doubling of urine Ba level was associated with a 57% increase in AKI diagnosis of any stage. Further studies are needed to study complement inhibition in treatment or prevention of AKI in critically ill children.

## Graphical abstract

**Figure:**
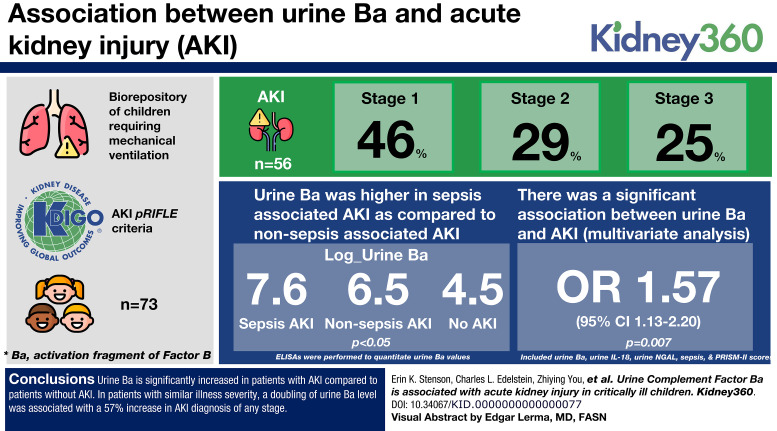

## Introduction

One in four children admitted to the pediatric intensive care unit (PICU) suffer from AKI, which is independently associated with increased morbidity and mortality. Severe AKI is associated with even higher mortality, morbidity, and resource utilization, including increased use of kidney replacement therapy, longer mechanical ventilation requirements, and longer length of stay in both the hospital and the intensive care unit.^1^ AKI survivors have worse health-related quality of life after discharge and are at high risk for chronic kidney disease.^2–4^ Other than supportive care, there are no treatment options which likely stems from the multifactorial etiology of AKI in critical illness, which includes hemodynamic perturbations, nephrotoxin exposure, and inflammatory dysregulation. These are all affected by the complement system, a component of the innate immune system.

Preclinical models of sepsis, ischemia reperfusion injury (IRI), and nephrotoxin-induced AKI clearly delineate that the complement system is instrumental in AKI pathogenesis.^5–10^ Complement has also been implicated in the pathogenesis of numerous primary kidney diseases, and there is emerging evidence of its role in AKI in critically ill adults and children.^11–16^ In adults with AKI after cardiac surgery, urine Ba (activated fragment of factor B, a component of the alternative complement pathway) increased proportional to AKI severity and before the rise in serum creatinine (sCr).^14^ In critically ill children, urine Ba increased as AKI severity increased.^15^ However, this pediatric study was limited by a small sample size and a single time point of complement measurements. The aim of this study was to determine the association between urine Ba and AKI development in an expanded cohort of critically ill children. We hypothesized that there was a difference between changes in urine Ba over time and AKI diagnosis. We further hypothesized that patients with sepsis-associated AKI (SA-AKI) would have the highest urine Ba values.

## Methods

### Patient Identification

Patients who were between ages 1 month and 21 years and admitted to the PICU at Texas Children's Hospital in 2006 were eligible for the original study of urine biomarkers.^17–19^ Additional inclusion criteria included receipt of invasive mechanical ventilation and/or vasoactive medications and presence of an indwelling bladder catheter.^17–19^ Patients with end-stage kidney disease or within 90 days after a renal transplantation were excluded. Patients were included in this study if they had at least one urine specimen available and at least two sCr measurements. The study protocol and consent were approved by Baylor College of Medicine Human Subjects Institutional Review Board before study initiation. The consent allowed for deidentified samples to be sent to University of Colorado where they were analyzed for urine IL-18 levels and subsequently stored until this study was performed.

### AKI Diagnosis

The original database was designed to study a modified pediatric version of the RIFLE (pRIFLE) criteria to describe AKI epidemiology and severity in critically ill children.^19^

pRIFLE AKI staging was based on urine output (UOP) or sCr and assessed daily. The original study assigned a baseline estimated creatinine clearance (eCCl) of 100 ml/min per 1.73 m^2^ to patients who did not have a baseline sCr.^17,19^ The pRIFLE criteria classifies patients according to the changes in UOP or eCCl, which was calculated using the Schwartz formula^20^—stage 1 (Risk): eCCl decreased by ≥25% or UOP <0.5 ml/kg per hour for 8 hours; stage 2 (Injury): eCCl decreased by ≥50% or UOP <0.5 ml/kg per hour for 16 hours; Stage 3 (Failure): eCCl decreased by 75% or UOP <0.3 ml/kg per hour for 24 hours or anuric for 12 hours.

### Specimen Collection

Patients were enrolled in this study on day 0 of invasive mechanical ventilation initiation. Daily urine was obtained for 4 days starting either on enrollment day or the following day. Urine Foley bags were emptied 1 hour before urine collection, and at least 5 ml of fresh urine from the urinometer was retrieved. Urine was kept on ice until centrifugation at 3000 RPM at 4°C for 5 minutes. The supernatants were aliquoted and stored at −80°C until analysis.

### Urine Ba Analysis

Levels of Ba and C5b-9 in urine were measured using commercially available ELISAs validated for clinical diagnostics (Quidel, San Diego, CA). Urine Ba values were measured on multiple days depending on biospecimen availability. The coefficient of variation of interassay reproducibility is 3.4% and for intra-assay reproducibility is 2.2%. A proportion of measurements were made in duplicate, and all were in a blinded fashion.

### Statistical Analysis

Baseline characteristics were compared among participants with and without AKI with the use of the chi-squared test for categorical variables, the *t* test for continuous variables, and the nonparametric test when appropriate. Chi-square testing was performed to investigate any differences in baseline characteristics between patients with available biospecimens and patients without available biospecimens. Mean urine Ba values were calculated based on stage of AKI and included all available urine Ba values for each patient. AKI etiology was divided into SA-AKI and non–sepsis-associated AKI (non SA-AKI). Sepsis was determined based on original investigators' discretion.

Additional analysis included day of urine acquisition relative to day of AKI diagnosis in four groups: samples in patients with no AKI, samples acquired in patients before AKI diagnosis (before AKI), samples acquired in patients on day of AKI diagnosis (day of AKI), and samples acquired in patients on days after initial AKI diagnosis (post-AKI). Urine Ba values were non-normally distributed and thus log-transformed before analysis.

To examine how a variable changed over time during the study period and its association with AKI development, data analysis using a mixed-effects model with random intercept and random slope was performed. In this analysis, patients with and without AKI within the study period were compared, no matter the time of AKI development. Days from enrollment was used as a continuous time in analysis, and the basic model included days, AKI status, and their interaction term. Multivariate logistic regression was performed using the binary outcome of AKI and no AKI. Logarithmic transformation to base 2 was performed on urine Ba values for inclusion in the regression analyses. Pediatric Risk of Mortality Scores-II (PRISM-II) were included to adjust for illness severity on admission to the PICU^21^ because PRISM-II was the proxy for illness severity used in the database from the original study.

## Results

### Patient Characteristics

There were 137 patients in the original study, and 73 patients had remaining urine specimens available for analysis. Patient characteristics are shown in Table 1. Seventeen patients had no AKI, and 56 patients had AKI of any stage: 26 patients with pRIFLE stage 1, 16 patients with pRIFLE stage 2, and 14 patients with pRIFLE stage 3. There were no significant differences in age, sex, PRISM-II score, PICU admission day enrolled, mortality rates, or sepsis in patients with AKI compared with patients without AKI. Comparing patients with available biospecimens with patients without available biospecimens found no difference in sex (*P*=0.31), age (*P*=0.19), day of AKI (*P*=0.26), IL-18 scores (*P*=0.84), mortality rates (*P*=0.25), or sepsis rates (*P*=0.15). In addition, proportions of patients without AKI and stage 1, 2, and 3 AKI were similar between the 72 patients with biospecimens included in the analysis and the 64 patients without biospecimens not included in the analysis (*P*=0.94).

**Table 1:** Characteristics of patients included in the study

Characteristic	Overall (*N*=73)	No AKI (*N*=17)	AKI (*N*=56)	AKI Stage		
				Stage 1 AKI (*N*=26)	Stage 2 AKI (*N*=16)	Stage 3 AKI (*N*=14)
Age (yr)^a^	3 (1, 12)	4 (1, 10)	2 (0.5, 12)	2 (0.3, 11)	2.5 (0.3, 12)	3 (1, 13)
PRISM-II score^a^	14 (7, 19)	14 (8, 18)	15 (7, 19)	15 (5, 20)	14 (11, 18)	14 (9, 19)
PICU admission day enrolled^b^	2 (2, 3)	3 (2, 3)	2 (2, 3)	2 (2, 30)	2 (2, 3)	2 (2, 3)
PICU AKI day^b^	1 (1,2)	n/a	1 (1, 2)	2 (1, 4)	1 (1, 2)	1 (1, 1)
Male (%)^b^	42 (58)	11 (65)	31 (55)	13 (50)	11 (69)	7 (50)
Mortality (%)^b^	13 (18)	3 (17)	10 (18)	3 (12)	6 (38)	2 (14)
Dialysis need (%)^b^	3 (4)	0	3 (5)	0	1 (6)	2 (14)
Sepsis (%)	12 (16)	3 (17)	9 (16)	1 (4)	5 (31)	3 (21)

PRISM-II score, Pediatric Risk of Mortality Score-II; PICU, pediatric intensive care unit; n/a, not applicable.

a *t* test used.

b Chi-squared test.

### Association between Urine Ba Values and AKI

**Figure 1:**
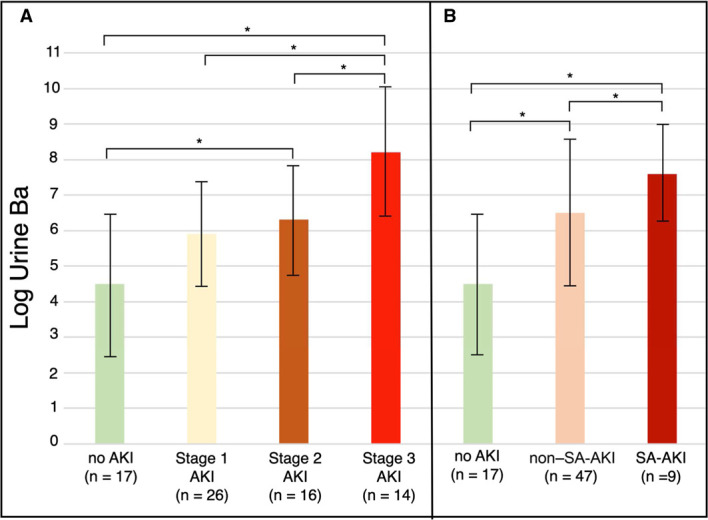
**Association between Urine Ba Values and AKI.** (A) Mean and standard deviation log values for pRIFLE AKI stages. (B) Mean urine log Ba values with interquartile range (IQR) for AKI due to sepsis (SA-AKI) compared with AKI not from sepsis (on SA-AKI). non–SA-AKI, non–sepsis-associated AKI; pRIFLE, pediatric version of the RIFLE criteria; SA-AKI, sepsis-associated AKI. *denotes significance at *P*<0.05.

Figure 1A shows the urine Ba level (mean±SD of log value) of patients with different AKI stages (as defined by pRIFLE) at day 1 of mechanical ventilation initiation. Urine Ba (log values) increased sequentially with worsening pRIFLE AKI stage (*P*<0.0001). When compared with patients with no AKI (4.5±2.1), patients with stage 2 AKI (6.3±1.6) had significantly higher mean log urine Ba values (*P*<0.05). Similar results were seen when comparing patients without AKI with patients with stage 3 AKI (8.2±1.9, *P*<0.05). In addition, there were significant differences between stage 3 AKI and stage 2 AKI (*P*<0.05) and stage 3 AKI and stage 1 AKI (5.9±1.9, *P*<0.05). Figure 1B shows the association between log urine Ba based on etiology of AKI. Patients with any stage SA-AKI had urine Ba values (7.6±1.4) than patients with non SA-AKI (6.5±2.1) and patients without AKI (4.5±2.1), *P*<0.00034.

### Mean Urine Levels Based on Pre-AKI, Day of AKI, and Post-AKI Diagnosis Specimen Acquisition

**Figure 2:**
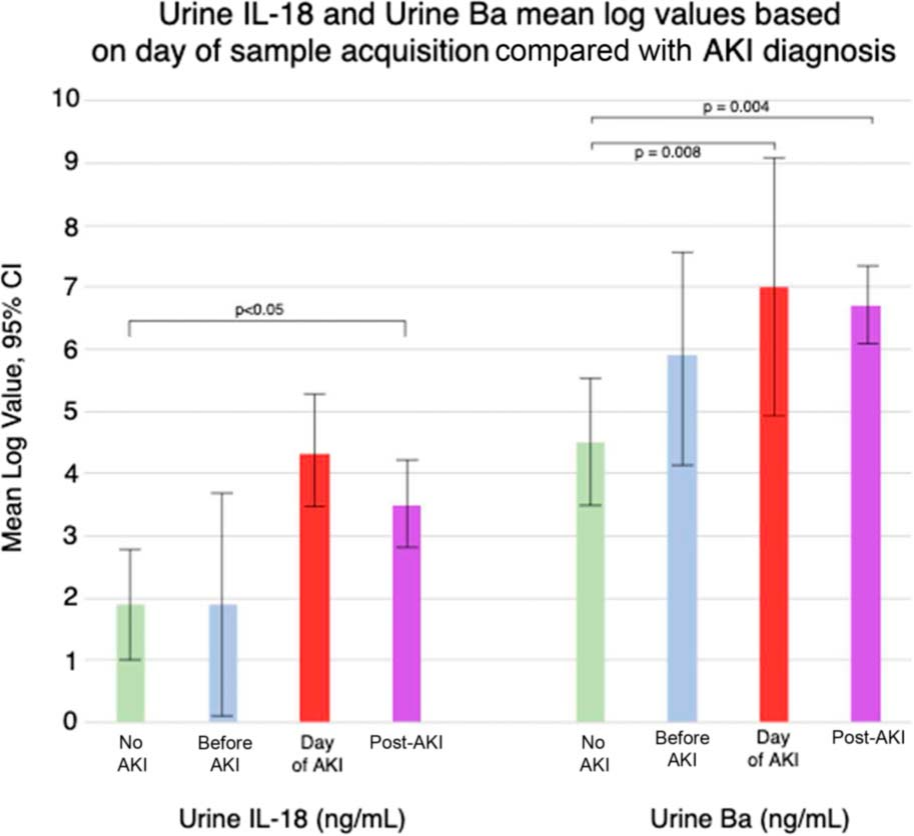
**Mean log values of urine Il-18 and urine Ba based on day of sample acquisition compared with AKI diagnosis.** CI, confidence interval.

Figure 2 shows mean log values of urine Ba and urine IL-18 based on day of sample acquisition compared with day of AKI diagnosis. Not all patients had samples available from each of these time points, and ultimately, there were 17 samples obtained in patients without AKI, nine obtained before AKI, seven on day of AKI diagnosis, and 40 on days post-AKI. Mean log urine Ba values were higher in subjects with values obtained on day of AKI diagnosis compared with values obtained from subjects without AKI (mean difference 2.5, 95% confidence interval [CI], 0.7 to 4.4; *P*=0.008). Similarly, mean log urine Ba values were higher in subjects with values obtained on days post-AKI diagnosis compared with subjects without AKI (mean difference 2.2, 95% CI, 1.0 to 3.4; *P*=0.0004). Compared with subjects without AKI, mean log urine IL-18 values were significantly higher in subjects with AKI on post-AKI days (mean 1.1, 95% CI, 0.06 to 2.1; *P*=0.04). There was no difference in mean log urine IL-18 values in specimens obtained on AKI day (mean 1.22, 95% CI, −0.29 to 2.52; *P*=0.12) or before AKI day (mean 1.05, 95% CI, −0.64 to 2.74; *P*=0.22) compared with subjects without AKI. We noted a trend of higher urine Ba values from samples obtained before AKI diagnosis compared with patients without AKI (*P*=0.10).

### Changes in Urine Ba and Urine IL-18 Based on AKI Diagnosis

With the mixed-effects model, we examined the pattern of how urine Ba (log value) changed over time during the study period. Patients with AKI had significantly higher estimated urine Ba than those without AKI on day of study enrollment (*P*=0.0042). Urine Ba did not change significantly throughout the study period in patients without AKI (*P*=0.46) whereas patients with AKI had a decrease in urine Ba values (*P* 0.049). The estimated difference in urine Ba between those with and without AKI reduced over time: day 1 (mean difference 1.8, *P*=0.0028), day 2 (mean difference 1.43, *P*=0.0043), and day 3 (mean difference 1.06, *P*=0.0301). There was no difference on day 4 (mean estimate 0.69, *P* 0.23). With the mixed-effects model, we evaluated how IL-18 (log value) changed over time and found a difference on day 1 (mean difference estimate 1.29, *P*=0.034) and day 2 (mean difference estimate 1.069, *P*=0.038).

### Comparison of Urine Ba, Urine C5b-9, Urine NGAL, and Urine IL-18

**Figure 3:**
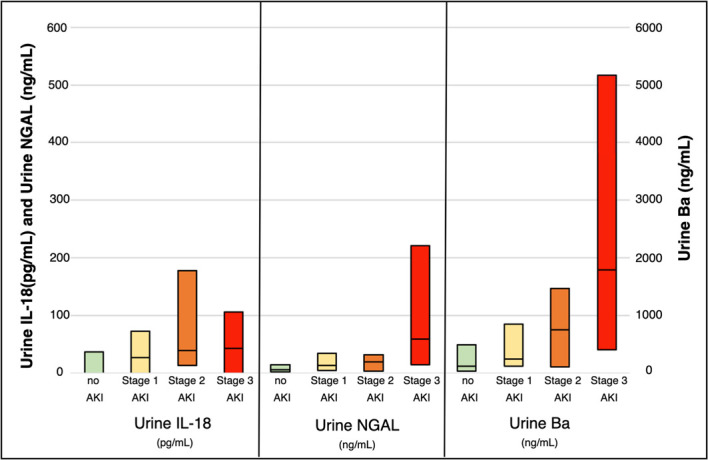
Increased urinary biomarkers (urine NGAL, urine IL-18, and urine Ba) as AKI stage increased.

Urine C5b-9 was analyzed in a random subset of samples. The fragments were degraded and not able to obtain values using ELISAS. The remaining samples were not analyzed for urine C5b-9 values. Urine Ba was compared with urine neutrophil gelatinase associated lipocalin (uNGAL) and urine IL-18 values, both of which are well-documented markers of kidney injury.^22–26^ Figure 3 shows similar patterns of elevation based on pRIFLE staging. Logistic regression was performed to test the association between urine Ba values and AKI diagnosis. In univariate analysis (Table 2), there was a significant association between urine Ba and AKI (odds ratio [OR] 1.46, 95% CI, 1.16 to 1.85, *P*=0.0015). Table 2 shows the multivariate analysis which included urine Ba, IL-18, uNGAL, sepsis, and PRISM-II scores. After multivariate adjustment, there was a significant association between urine Ba and AKI (OR 1.57 [95% CI, 1.13 to 2.20]; *P*=0.007) and urine NGAL and AKI (OR 1.57 [1.12 to 2.20]; *P*=0.009), but not urine IL-18.

**Table 2:** Univariate and multivariate logistic regression testing for an association between urine Ba and AKI

Variable	OR (95% CI)	*P*-value
**Univariate analysis**		
Log urine Ba	1.46 (1.16 to 1.85)	0.0015
**Multivariate analysis** ^a^		
Log urine Ba	1.57 (1.13 to 2.20)	0.007
Log urine IL-18	1.06 (0.83 to 1.35)	0.66
Log urine NGAL	1.57 (1.12 to 2.20)	0.009
Sepsis	0.13 (0.017 to 1.064)	0.057
PRISM-II	0.99 (0.90 to 1.09)	0.91

OR, odds ratio; CI, confidence interval; PRISM-II, Pediatric Risk of Mortality Score.

a Adjusted for sepsis, urine IL-18, urine NGAL.

## Discussion

This is the first study examining urine complement factor B fragments in patients with and without sepsis in a moderately sized cohort of critically ill children. Urine Ba increased in a stepwise fashion as AKI severity increased and was highest in patients with SA-AKI. This stepwise increase mirrored other established AKI urinary biomarkers, such as NGAL and IL-18. After adjusting for illness severity, urine Ba and urine NGAL were associated with AKI diagnosis. In patients with similar illness severity on admission, a doubling of urine Ba level was associated with a 47% increase in AKI diagnosis. Urine Ba may be both a mediator and a biomarker of AKI, providing a potentially novel intervention opportunity because complement-targeted therapeutics is used safely and effectively to treat other diseases.

Our findings add to prior preliminary evidence that originally demonstrated the association between urine Ba and AKI severity. Two pilot studies, one in pediatric critically ill children and one in adults after cardiac surgery, were the first to identify the independent association between urine Ba values and AKI severity.^14,15^ Secondary analysis of this pilot study also showed that urine Ba value was significantly increased in patients with SA-AKI compared with non–SA-AKI.^27^ Furthermore, multivariate analysis showed that urine Ba and urine NGAL had similar ORs for AKI, highlighting the potential for urine Ba to perform similarly to urine NGAL as an AKI biomarker.^22,26,28–32^ Using urine Ba as an AKI biomarker has the important distinction that a small molecule factor B inhibitor is currently in clinical trials for other complement-mediated diseases.^33,34^

Together with the preclinical studies, there is now increasing evidence that implicates the alternative complement pathway in AKI pathogenesis, especially after nephrotoxin exposure, bilateral IRI, and sepsis.^5,7,8,10,35^ Complement factor B is pivotal given its role in the feedback amplification loop. When the alternative pathway is activated, factor B is cleaved by Factor D into two activation fragments: Ba and Bb. Bb combines with C3b to form the C3 convertase (C3bBb) which converts new molecules of C3 into the anaphylatoxin C3a and C3b. Each resulting C3b can combine with additional Bb to create more C3 convertases, and thus, the feedback amplification loop leads to further complement activation. C3b and Bb also combine to create the C5 convertase which converts C5 into C5a (another anaphylatoxin) and C5b. C5b joins with C6–C9 to create C5b-9, also known as the membrane attack complex, or the terminal complement complex.

It is plausible that increased levels of factor B lead to increased generation of anaphylatoxins (C3a and C5a) and the membrane attack complex. The anaphylatoxins trigger release of proinflammatory mediators and vascular leak from altered vascular permeability.^36^ The membrane attack complex lyses target cells; this, while beneficial for pathogen clearance, can also damage host tissues.^11^ Together the anaphylatoxins and membrane attack complex may offer insight into why there is significant tissue inflammation with AKI even after nonimmune insults such as IRI and nephrotoxin exposure. This inflammatory process seems to be attenuated in mice with targeted genetic deletion of Factor B (fB^−^/^−^). In mouse models of IRI, fB^−^/^−^ mice had attenuated kidney tissue damage and lower AKI biomarkers in addition to minimal C3 and c5b-9 deposition—implying that complement activation after IRI occurs through the alternative pathway exclusively.^8^ In a sepsis model, fB^−^/^−^ mice had improved survival, attenuated kidney tissue damage, reduced proinflammatory cytokines, and retained ability to clear infection.^7^ Importantly, wild-type mice treated with a direct Factor B inhibitor had similar findings in both IRI and sepsis models.^5,35^ With this preclinical evidence implicating Factor B and our clinical observational studies showing the association with urine Ba and AKI, there is increasing motivation for future clinical studies evaluating urine Ba and trialing complement-targeted therapeutics. These clinical trials should ideally be focused in patients with sepsis given the finding showing the highest urine Ba in SA-AKI.

Our study has some limitations. This was performed *post hoc* and performed on urine samples that had been collected 15 years before urine Ba analysis. However, the samples had been stored and frozen appropriately, and the pattern of urine Ba elevation was consistent with prior reports, and the pattern of elevation was consistent with previously analyzed urine biomarkers, such as IL-18 and NGAL.^17,18^ We believe that the age of the samples affected our ability to measure urine C5b-9, which was consistent with previous studies.^14^ These reports hypothesized that urine sC5b-9 had shorter half-lives, lower stability in urine, or decreased sensitivity on ELISAs to detect compared with urine Ba fragments. There were limited number of control patients without AKI, and AKI was diagnosed based on the pRIFLE criteria. While current AKI diagnosis is based on the Kidney Disease Improving Global Outcomes criteria, pRIFLE has similar cutoffs and both are based on changes in sCr. Irrespective of the AKI definition used, the association between AKI and ICU related morbidity and mortality remains concerningly high.^37^ Compared with more recent use of an eCCl of 120 ml/min per 1.73 m^2^, we used a lower eCCl of 100 ml/min per 1.73 m^2^ and may have underestimated AKI staging of our patients. However, the overall trend of our findings is similar to prior work and shows that AKI staging correlates with urine Ba levels. Finally, although there was a trend that showed urine Ba levels before AKI were higher than those without AKI, not enough biospecimens were available to determine the ability to use urine Ba to predict future AKI.

Our study also has multiple strengths. This is the first study to compare urine Ba with other urinary biomarkers of kidney damage in critically ill children. Despite the age of the biospecimens, we were able to analyze them for factor B, unlocking future research opportunities on complement activation fragments from other biorepositories. Finally, our findings are consistent with studies of smaller pilot populations and further solidify the association with urine Ba and AKI severity and urine Ba in SA-AKI.

In summary, we found that urine Ba increases as AKI severity increases. Larger studies with additional samples obtained before AKI diagnosis are needed to determine the utility of urine Ba as a predictive biomarker for future AKI development. Specific studies in the sepsis population are also needed to determine the association between urine Ba and AKI severity within this population. Urine Ba also shows potential for prognostic enrichment in future clinical trials evaluating AKI treatments or enhanced supportive care modalities. In addition, severe elevation of urine Ba may identify critically ill patients who would benefit from factor B inhibition. Overall, our study calls for increased investigation into complement-mediated AKI in critically ill patients with specimen acquisition before AKI diagnosis.
